# Trends analysis for drug utilization in county public hospitals: a sample study of the pilot area of health care reform in China

**DOI:** 10.1186/s12913-018-3614-8

**Published:** 2018-10-23

**Authors:** Xuefeng Xie, Xu Jin, Ling Zhang, Huihui Sun, Aizong Shen, Xiaohui Huang, Yehuan Sun

**Affiliations:** 1 Department of Basic and Clinical Pharmacology, Anhui Institute of Innovative Drugs, School of Pharmacy, Anhui Medical University, Hefei, 230032 Anhui China; 2 Department of Epidemiology and Health Statistics, School of Public Health, Anhui Medical University, Hefei, 230032 Anhui China; 3 Department of Pharmacy, Anhui Provincial Hospital, Hefei, 230001 Anhui China

**Keywords:** Drug utilization, Health care reform, Rational use of medicine, Pharmacovigilance

## Abstract

**Background:**

Changes in the national drug policy always have impact on the drug utilization. In the context of China health care reform, what changes had happened in the trend of drug utilization in public hospitals? Has this change met the expectations of policy design? This study was conducted to explore the trend of medicine consumption in county public hospitals before and after health care reform, and to provide real-world evidence to help assess the effectiveness of national drug policy.

**Methods:**

A cross-sectional study was performed to investigate the drug utilization trends of 6 county public hospitals in Anhui Province, which is the first pilot area of China health care reform. Data were collected before and after the implementation of the China National Essential Medicine Policy (NEMP) to analyse the drug utilization indicators, such as the drug utilization constituent ratio, the rate of essential medicine usage and the rate of antibiotic consumption.

**Results:**

Chemicals are used most frequently and account for 60%~ 70%, followed by oral agents of proprietary Chinese medicine. The results also show increased consumption of Chinese medicine injections (*χ*^*2*^ = 28.428, *P* ***<*** 0.01). The top 3 chemical medicines consumed were anti-infective drugs (12.92%), cardiovascular system drugs (11.61%), and digestive system drugs (8.42%). For Chinese traditional medicine, the top 3 drugs consumed were internal medicine drugs (66.03%), surgical drugs (8.45%), and gynaecological drugs (7.70%). The total sales amounts of drugs covered by medical insurance are at a high level (all above 80%), whereas essential medicines are less than 50% at almost all county-level medical institutions.

**Conclusions:**

This study uncovered the changing tendency of medicine usage under the implementation of the reform. Chinese medicine injections and anti-infective drugs have always been a sustained concern of pharmacovigilance. It is noteworthy that although essential medicines are advocated for as a priority for use in the government-run hospital, the consumption proportion of these medicines is lower than expected.

## Background

Promoting the rational allocation of medicine resources and ensuring drug safety application should be the essential goal of health care reform regardless of whether reform occurs in developing countries or developed countries. Since 2009, China has been pushing forward with a new round of the phased implementation of health care system reform centred on the China National Essential Medicine Policy (NEMP) [1]. The reforms focus on establishing public medical insurance systems and enhancing accessible and affordable public healthcare services and medicines.

In urban and rural areas, three government medical insurance systems, Urban Residents Basic Medical Insurance, Urban Employee Basic Medical Insurance and New Rural Co-operative Medical Scheme provide medical benefit to urban workers, residents and farmers and pave the way for universal health insurance. However, with the rising cost of medical attention, NEMP was introduced as a core element of China’s healthcare system and policy reform, aiming to provide standard and basic medication supply security to each individual. There has been a great change in the system of preferential use of essential medicines in hospitals, zero-profit sales and centralized bidding and purchasing of medicines, which have a great impact on the hospital pharmacy delivery model.

China’s public health institutions are managed in a hierarchical model composed of primary, secondary and tertiary medical institutions; this system is also a focus of overall health care reform. China health care system reform has been piloted from the primary hospitals and gradually implemented in county hospitals (the secondary medical institutions) and comprehensive tertiary hospitals. With the thorough implementation of medical reform throughout the country, the importance and complexity of the reform of comprehensive senior hospitals has been highlighted. County public hospitals are the main medical institutions that provide health service for urban and rural populations. They have become important links from the reform of primary medical institutions to the overall implementation in China. Have there been any significant changes in the composition of drug use? If so, what is the trend? These are all issues that should be of concern. Although there are many factors influencing the final results of medicine consumption, the dynamic changes in the basic composition of drug utilization could still provide much useful information for decision-making, which can better guide clinical rational medication. To help provide evidence for an assessment of NEMP, a consistent drug utilization review should be conducted in secondary and tertiary hospitals.

Anhui Province, which is located in the middle east of China, is the earliest province to implement a zero-profit sales policy for medicines in primary hospitals and is also a pilot province in China health care reform. As of December 2013, there were 730 public hospitals in Anhui Province, and 274 were secondary hospitals, with a total population of nearly 60 million permanent residents in the province. Based on the requirements of health care reform, “To separate the benefits of medicine from treatment, perfect the public hospitals compensation mechanism” with the purpose of understanding the baseline data of drug utilization, identifying the breakthrough point of drug supply for county hospitals and providing references for the formulation of drug policies. We adopted a qualitative and quantitative investigation to evaluate the current situation regarding the burden of disease and medicines utilized in county medical institutions, aiming to provide decision-making guidelines and methodological structure to ensure the rational use of medicines.

## Methods

### Sample selection

To ensure the representativeness of the samples, subject-stratified sampling was conducted in three districts of the North, Middle and South areas of Anhui Province based on the characteristics of economic and administrative divisions in Anhui Province; one county public hospital and one county traditional Chinese medicine hospital were selected in each city. In 2009, the Chinese government selected Anhui, along with a couple of other regions, for a pilot launch of the NEMP in primary healthcare institutions. In 2012, NEMP was gradually implemented in county hospitals (the secondary medical institutions) in Anhui province. So, drug utilization data were extracted from 2011 through 2013, the three consecutive years before and after the implementation of NEMP in Anhui province.

The field investigation was conducted during December 2015 to April 2016. All of the involved hospitals signed a data use agreement, and three calendar years were set: January 1, 2011 ~ December 31, 2011, January 1, 2012 ~ December 31, 2012 and January 1, 2013 ~ December 31, 2013. These data were exported from the Hospital Information System (HIS) by the required character segments included drug unit price, variety and specification information, and amount of drug sales, and the database of drug utilization was then established. At the same time, critical persons interviews were used in qualitative analysis to understand the status of implementation of the essential medicine system, medical reimbursement, and the structure of healthcare providers. Interviewees included hospital managers, clinicians and pharmacist representatives.

### Drug utilization review

The trends of drug utilization were evaluated in terms of three sets of outcomes. The first was the change of overall drug utilization constitution which was classified by pharmaceutical dosage forum and pharmacological action. The second was the anti-infective medicine utilization analyse which was summarized by calculating two variables, category quantity and amount of sale. The third, in order to figure out whether essential medicines were used preferentially in county hospitals, percentage of essential medicine utilization was calculated and compared with National Basic Medical Insurance Medicine. The distribution of category quantity and sale of different medicine list was highlighted.

Drug constituent ratios of quantity and expense were analysed as well as the utilization of the National Essential Medicine List and the Basic Medical Insurance Medicine List. The major indicator analysis methods are as follows.

Constituent ratio of drug category quantity: The constituent ratio of category quantity was used to describe the general drug use condition and to calculate the proportion of actual drug use in medical institutions in the two medicine lists.

Constituent ratio of drug sales: The total amount of the single drug used by a medical institution was calculated, which can be used to compare the consumption level of pharmaceutical resources in different regions. At the same time, the total sales of various drugs were sorted and numbered to obtain the sales amount order of each drug.

### Statistical methods

The data volume is large since it includes the sources of information on the consumption of pharmaceuticals and interview data from six county public hospitals for 3 years. Therefore, the research group is divided into three groups according to the 3 sample counties. A team leader responsibility system was set up, and each group has two researchers for data entry. Cross examination was conducted on 10% of the total quantity of drug varieties. Statistical Package for the Social Sciences (SPSS) 17.0 statistical software was used to analyse data, including drug category, category quantity, frequency of use and amount of sale. Among them, the statistical description of count data mainly adopts the constituent ratio indicator, and the description of measurement data is represented by $\overline{x}$±s.

## Results

### Theme 1: Changes in the overall constitution of drug utilization

Because of the data-use confidentiality agreement, “County A” represents samples in the Middle Anhui Province, “County B” represents samples in the Northern Anhui Province, and “County C” represent samples in the Southern Anhui Province. According to the analysis of the constitution of drug utilization classified by pharmaceutical dosage forms, the results showed a significant difference in varieties dosage form of drugs when comparing the three regions in the 3 years (2011 to 2013). The drug varieties used in the six county hospitals, excluding decocting pieces of Chinese medicine, ranged between 575 and 4037 (drugs of different dosage forms and specifications were listed separately, which mainly focused on the reality of drug use), which may be related to the distinct demographics and economy of each city.

**Fig. 1:**
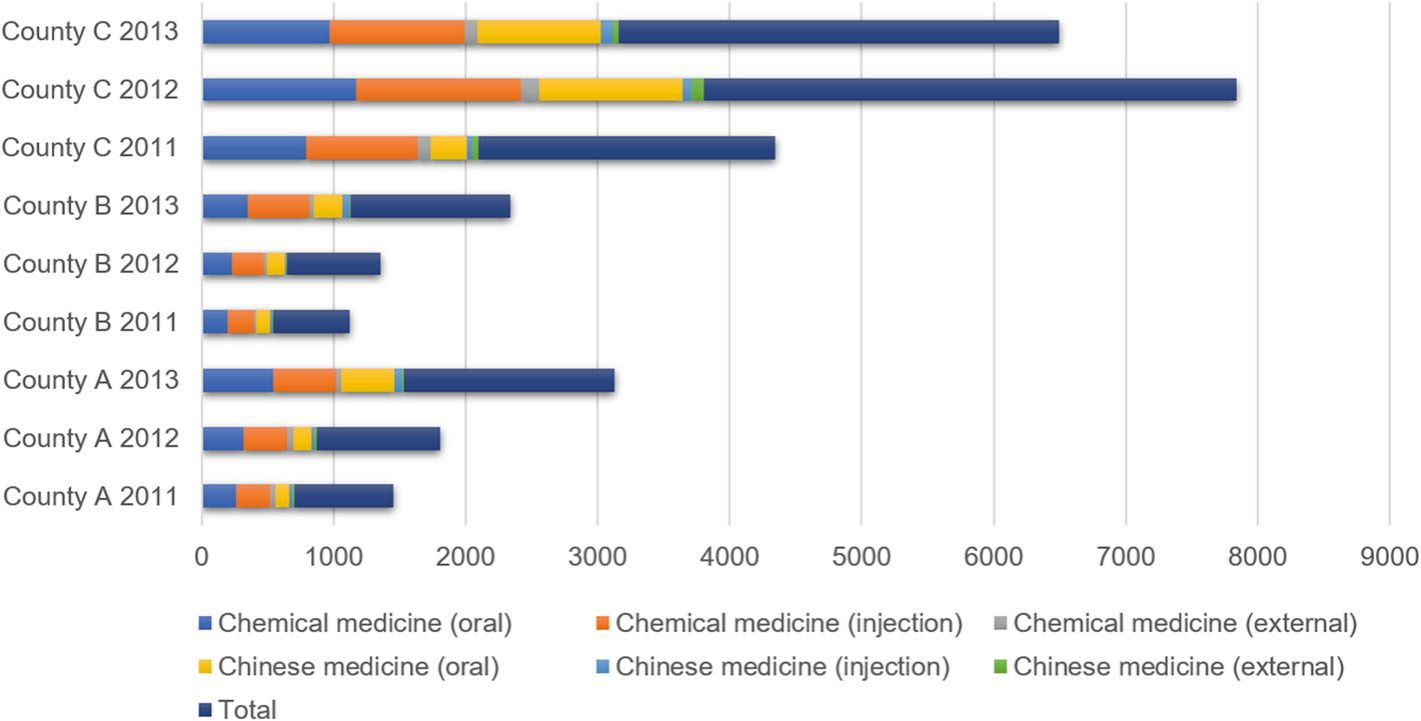
Category quantity in each county from 2011 to 2013 (counted by pharmaceutical dosage forms)

As shown in Fig. 1 and Table 1, the category quantity of chemical medicines used in Counties A, B, C is relatively high and constitutes approximately 60% ~ 70%, including oral agents and injections, followed by proprietary Chinese medicine oral agents.

**Table 1:** Constituent ratio of different dosage form of drugs consumption in the sample area from 2011 to 2013

Year	Chemical medicine (oral %)	Chemical medicine (injection %)	Chemical medicine (external %)	Chinese medicine (oral %)	Chinese medicine (injection %)	Chinese medicine (external %)	Others (%)	*χ* ^*2*^	*P*
2011	1251 (35.00)	1300 (36.41)	154 (4.31)	493 (13.81)	70 (1.96)	79 (2.21)	223 (6.30)	243.779	0.00
2012	1716 (30.23)	1819 (32.04)	209 (3.68)	1354 (23.85)	107 (1.88)	127 (2.24)	335 (6.08)		
2013	1860 (30.27)	1966 (32.00)	166 (2.70)	1568 (25.52)	188 (3.06)	79 (1.29)	317 (5.16)		

Statistical analysis showed that there was a significant difference in the varieties of drugs consumed in the 3 years before and after the implementation of the reform (*χ*^*2*^ = 243.799, *P* ***<*** 0.01) (Table 1). Concurrently, we also focused on the pharmacovigilance issues of Chinese medicine injections, which have increased in utilization during the 3 years and show a statistically significant difference (*χ*^*2*^ = 28.428, *P* ***<*** 0.01).

### Theme 2: Drug utilization analysis classified by pharmacological action

Based on classification methods used in the National Essential Medicine List, chemical drugs were classified according to the pharmacological action, and traditional Chinese medicines were classified according to function. In addition, the drug utilization data of three consecutive years in 3 sample counties were classified and statistical analysed. The results show that the top three chemical drugs with the highest specification constituent ratio were anti-infective drugs (12.92%), cardiovascular system drugs (11.61%) and digestive system drugs (8.42%). For proprietary Chinese medicine, the top three were internal medicine (66.03%), surgery (8.45%) and gynaecological medicine (7.70%) (Table 2).

**Table 2:** Constituents in sample areas from 2011 to 2013 and counted by pharmacological action and function

Medicine	2011			2012			2013			Constituent Ratio (%)
	A	B	C	A	B	C	A	B	C	
Chemical Medicine										
respiratory system agents	27	15	37	33	20	44	55	35	68	4.11
anti-inflammatory drugs	24	37	62	31	43	90	57	57	116	6.36
psychotropics	11	10	17	13	13	20	18	18	25	1.78
anti-infectious agents	86	85	113	94	82	132	147	114	197	12.92
anti-allergic agents	8	7	16	9	7	17	13	11	23	1.36
anti-parasitic agent	2	2	5	2	2	6	1	2	6	0.34
anti-neoplastic	25	6	24	33	14	31	43	21	42	2.94
narcotics	14	14	28	17	16	31	22	24	46	2.61
urinary system agents	10	3	13	11	8	17	27	15	30	1.65
immunomodulators	10	5	19	18	5	10	19	11	22	1.46
other drugs	24	56	114	36	68	120	37	95	154	8.66
endocrine system agents	47	26	66	52	33	79	85	48	116	6.79
nervous system agents	36	30	69	49	34	72	71	73	134	6.98
biological	4	2	2	4	2	5	7	3	3	0.39
saccharides, salts, acid-base balance adjuvant	31	25	53	35	28	56	60	59	103	5.53
Vitamins, minerals and trace elements	24	20	54	26	21	52	41	32	73	4.22
digestive system drugs	45	38	83	57	46	90	101	78	147	8.42
cardiovascular agents	69	53	114	86	64	125	137	106	190	11.61
blood system agents	26	25	50	33	26	55	48	45	77	4.73
nutritional therapy agents	8	12	12	10	12	12	18	20	20	1.52
analgesics	14	0	26	22	0	0	26	0	0	1.08
specialist drugs	35	0	0	45	0	0	54	0	0	1.65
Chinese Medicine										
internal medicine drugs	91	2	223	122	106	798	353	202	744	66.03
other drugs	1	78	11	3	3	28	6	3	24	3.93
gynaecological drugs	15	8	29	16	20	92	35	20	73	7.7
surgical drugs	8	10	38	10	13	62	42	18	137	8.45
orthopaedics drugs	13	12	41	13	23	87	14	23	52	6.95
otolaryngology drugs	7	4	16	7	6	37	9	14	15	2.88
paediatric	0	2	0	0	3	0	0	3	0	0.2
ophthalmology	1	1	6	2	1	21	3	2	13	1.25
oncology	11	2	8	12	3	19	23	13	14	2.63

Note: In accordance with the “Notice for clinical medication of the People’s Republic of China Pharmacopoeia” 2010 edition classification, the other drugs include disinfection, antisepsis of chemical medicine, antidote, X-ray imaging and diagnostic medicine; obstetrics and gynaecology medication; and dermatologist, oculist, otolaryngology and dental and paediatric drug use. The other drugs of Chinese medicine include those for dermatology and stomatology

### Theme 3: Anti-infective medicine utilization analyses

From the perspective of the overall composition of 3 years of drug utilization, it was determined that anti-infective medicines are always the most-used. In addition, there are many different kinds of anti-infective drugs and the same kinds of anti-infection drugs have a variety of specifications, which inevitably increase the difficulty of the reasonable use of anti-infectives. In this context, there has been a gradual increase in drug-resistant strains resulting from the unreasonable use of anti-infective drugs, which has captured global attention. The normative use of the Essential Medicine List provides a feasible approach to rationally guide clinical drug use. The results of this study regarding the utilization of anti-infective drugs in county medical institutions in the sample counties are as follows.

#### Amount of anti-infective medicine sales

The results show that the amount of sales of anti-infective medicines increased between 2011 and 2013, but there is no significant difference in the proportion of the total sales amount (***χ***^***2***^ = 0.526, *P* = 0.971) (Table 3). Combined with the results of field interviews, we found that the application of the clinical hierarchical restriction on anti-infective medicines has gradually become standardized in recent years.

**Table 3:** Amount of anti-infective medicines’ sales and their percentage in total amount from 2011 to 2013 in each county

Item	2011			2012			2013		
	A	B	C	A	B	C	A	B	C
Medicine categories	86	85	113	94	82	132	147	114	197
Amount (thousands)	3530.3	11,419.9	1237.5	16,557	14,347.1	6946.8	15,365.6	11,955.5	18,263.3
Amount percentage (%)	32.13	35.4	18.84	31.22	33.06	15.74	31.95	31.33	20.49

#### Categories quantity of anti-infective medicine

The results show that anti-infectives used in county public hospitals in the three sampling counties in 2011~ 2013 increased year by year. Cephalosporins, penicillin, and anti-viral drugs account for more in the total consumption quantity over the 3 years, and broad-spectrum penicillin and other β-lactam antibiotics are primarily used. The number of categories of sulphonamides and tetracycline anti-infective drugs was essentially 0 (Table 4). The advantages of penicillin, which is included in the National Essential Medicine List, include a strong antibacterial effect, a high curative effect, low toxicity and a low price. Clinicians have better comprehension of such drugs, and patients have higher drug compliance. These factors explain the wide use of penicillin products in clinical treatment. In contrast, the number of categories for amino glycosides, chloramphenicol, tetracyclines and sulpha four anti-infective drugs are less than 10.

**Table 4:** The category quantity and constituent ratio of anti-infective medicine in the sample areas from 2011 to 2013

Year	2011			2012			2013			Constituent ratio (%)
	A	B	C	A	B	C	A	B	C	
Cephalosporin	23	17	32	22	23	35	35	37	40	25.14
Quinolones	6	5	5	8	5	8	11	5	18	6.76
Macrolides	8	4	6	9	4	10	14	8	15	7.43
Penicillin	21	19	23	20	15	23	36	16	29	19.24
Narrow-spectrum	3	2	4	4	3	2	4	1	3	2.48
Broad-spectrum	15	10	16	13	7	15	26	10	20	12.57
Other β-lactams	3	7	3	3	5	6	6	5	6	4.19
Aminoglycosides	2	5	4	2	4	4	2	2	4	2.76
Lincomycin	3	2	2	3	2	3	5	5	3	2.67
Chloramphenicol	0	1	1	0	1	1	0	2	1	0.67
Sulphonamides	0	1	1	0	1	1	0	1	2	0.67
Tetracyclines	0	0	1	0	0	1	0	0	1	0.29
Antivirus	7	8	12	9	8	18	20	17	28	12.1
Others	16	23	26	21	19	28	24	21	56	22.29
Total	86	85	113	94	82	132	147	114	197	100

Note: Penicillin includes narrow-spectrum penicillin, broad-spectrum penicillin and other β-lactams and other classes include anti-tuberculosis, ornidazole and others

The growth rate for the numbers of categories is more obvious in cephalosporins, followed by macrolides, antivirals, penicillin and quinolones. Their generalities are broad-spectrum, resistant to enzymes, moderate in price, easy to use, effective in treatment and show less adverse reactions. Some drugs, such as chloramphenicol and aminoglycosides, did not show an increase in the number of medicine categories. It is essential to note that the use of antimicrobial drugs in hospitals at all levels has been gradually standardized since the nation started the nationwide remediation of antimicrobial drugs in 2011 [2–4]. However, we were still able to draw a conclusion from Table 4: the usage of broad-spectrum antibiotics is much higher than narrow-spectrum antibiotics and other anti-infectives, such as antiviral drugs (12.10%), which require standardization along with further pharmacovigilance monitoring.

The category quantity of tetracyclines, sulphonamides and chloramphenicol in anti-infective drugs that are used is relatively small. The main consideration for less utilization of aminoglycosides is their ototoxicity and nephrotoxicity. The main adverse reactions of tetracycline drugs are damage to the reproductive system, the blood system, and the growth of the bones and teeth of children. Chloramphenicol may cause aplastic anaemia and grey infant syndrome. Therefore, the clinical use of these drugs, especially in children, lactating women, the elderly and patients with liver and kidney dysfunction needs therapeutic drug monitoring. At the county level hospitals, the equipment and pharmacy services for treatment and clinical monitoring of medication therapy still demand improvements. In this context, the use of these drugs requires strict indications.

### Theme 4: Tendency of essential medicine utilization

#### Categories quantity analyses

**Fig. 2:**
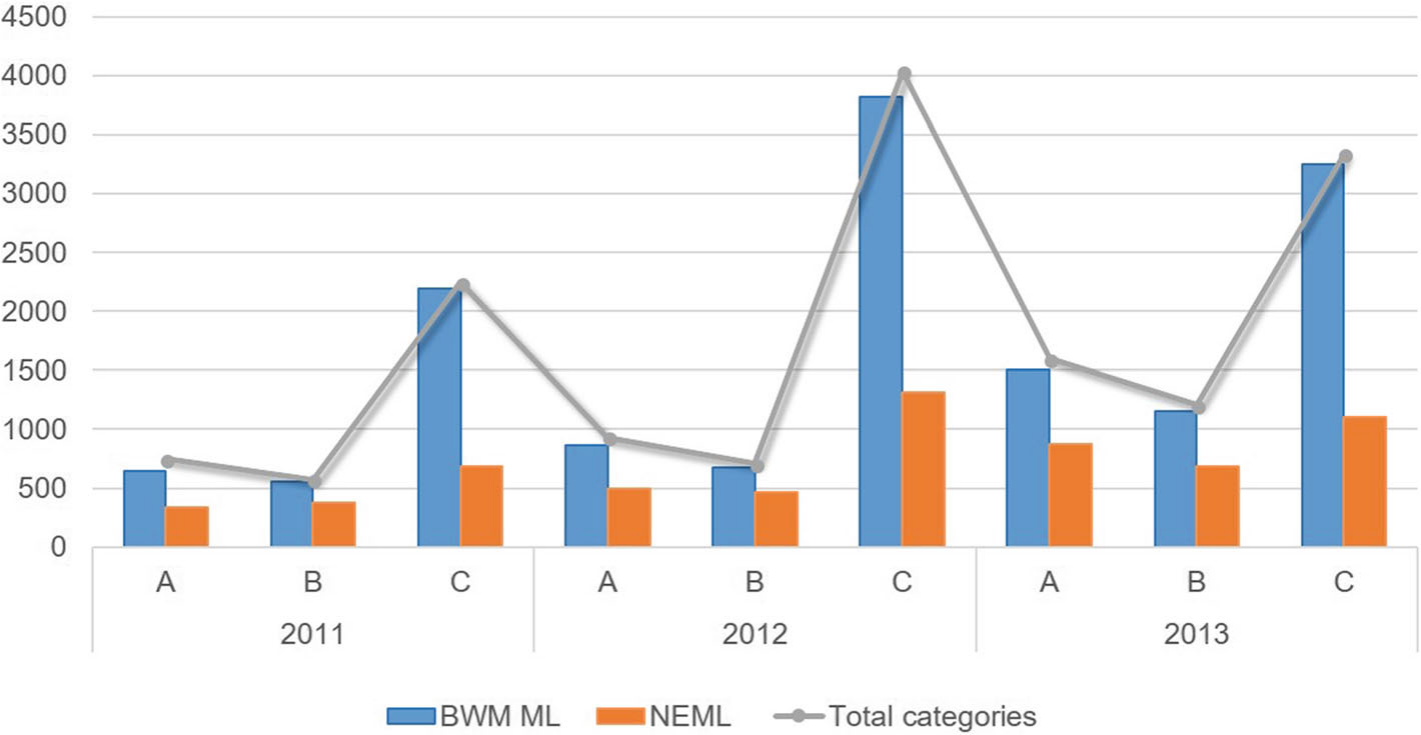
Constituent ratio of BWM ML and NEML used in the sample areas from 2011 to 2013 (calculated by categories quantity)

According to the “National Essential Medicine List” (NEML, 2009, 2012 edition) and the “National Basic Medical Insurance, Work-Related Injury Insurance and Maternity Insurance Medicine List” (BWM ML, 2011 edition), the percentages of BWM ML and NEML used during 2011~ 2013 in the Anhui county medical institutions that were sampled were calculated (Fig. 2). The results show that during this three-year period, the proportion of medicines used in medical insurance was high, and the highest proportion of medicines used in medical insurance was 97.73% in County C in 2011 (the percentage of essential medicines in the same period is 30.68%). The proportions of essential medicines are all lower than the medicines used by medical insurance.

To further clarify differences in the usage of the medicine lists in different years, we conducted a comparative analysis of the constituent ratio of the essential medicine list and basic medical insurance medicine list among 2011, 2012 and 2013. Chi-square analysis showed that there was no significant difference in the constituent ratio of drugs used in NEML and BWM ML between 2011 and 2012 (BWM ML: ***χ***^***2***^ = 1.069, *P* = 0.301; NEML: ***χ***^***2***^ = 1.324, *P* = 0.250). However, for the comparative analysis of 2011 and 2013 and the comparative analysis of 2012 and 2013, it was observed that there was a significant difference in the constituent ratio of the medicine lists from the 2 years (*P* < 0.001).

#### Amount of sales analyses

**Fig. 3:**
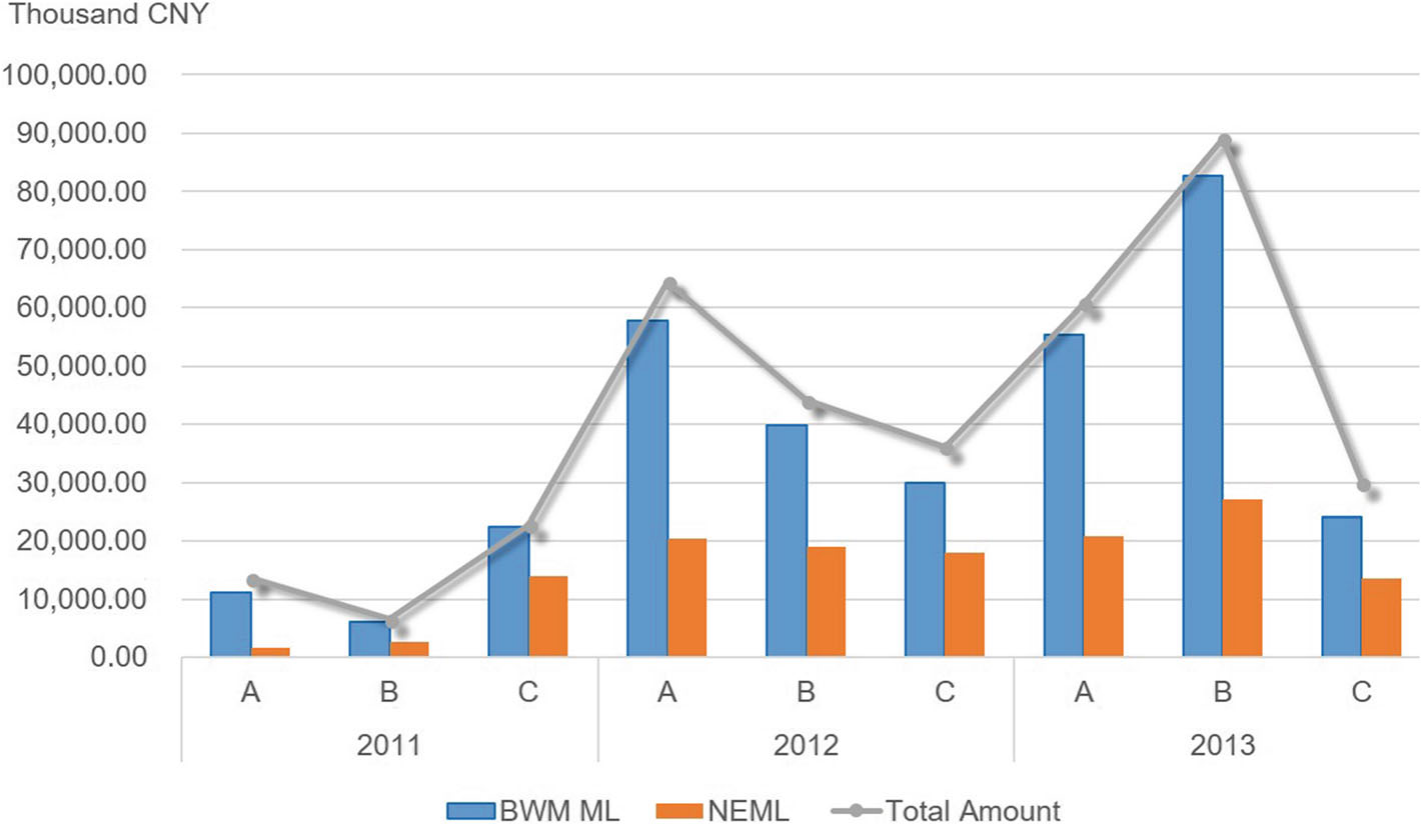
Constituent ratio of BWM ML and NEML used in the sample areas from 2011 to 2013 (calculated by amount of sales)

When calculating the proportion of the medicine list from the perspective of the drug consumption amount, it was determined that the total sales of drugs in BWM ML, which are all above 80%, account for a relatively high level of the total sales, whereas the sales of NEML only accounts for less than 50% of the total sales in all county medical institutions (only County C reached 61.53% in 2011). The BWM ML is used more frequently, and its proportion of sales is relatively high, which shows that the implementation of the basic medical insurance system has made promising progress in county medical institutions (Fig. 3).

Similarly, to pinpoint the differences in the two lists in terms of sales amounts in different years, a comparative analysis was applied on the constituent ratio of NEML and BWM ML in 2011, 2012 and 2013. Chi-square analysis results show that in 2011 vs. 2012, 2011 vs. 2013, and 2012 vs. 2013, the proportions of the sales amount in each group in both the NEML and the BWM ML have significant differences (*P* < 0.001).

Anhui Province is the first pilot areas (since September 2010) in China to implement a non-profit policy on the sales of essential medicines. At the end of 2011, it launched the comprehensive reform of county public hospitals. In October 2012, Anhui made the “Essential Medicine List for County Hospitals” from the “National Essential Medicine List,” the “New Rural Cooperative Medicine List” and the “Medicine List of Urban Medical Insurance”, and this list has been recommended for use in the unified bidding procurement for county hospitals [5]. In January 2013, all of the drugs sold in the 148 county hospitals of the 74 pilot cities across Anhui Province started to carry out the non-profit policy. County medical institutions also incorporate the control of drug structure as one of the performance appraisals for the purpose of strengthening the management of drug abuse. In this context, both the changes in the use of drugs and the changes in the price mechanism of medical institutions will result in significant differences in the amount of drug sales.

## Discussion

In the past 10 years, drug consumption in almost all countries throughout the world has been rapidly increasing. This has resulted in widespread concern in the public, and therefore, researchers have focused attention on studies related to drug sales, the drug consumption structure, factors affecting drug consumption, prescription habits, household medicines and related research [6], especially when the national drug policy changed. Since 2009, China has promoted a deepening reform of the medical and healthcare system to provide the public with equal access to medical and health resources [1]. One of the main components of the reform is a major approach to improve the drug supply security system to establish a national essential medicine system. Changes in national drug policy will inevitably result in the redistribution of resources of various stakeholders in the drug industry and will have an impact on the actual use of clinical medicines. Under the background of medical reform, an important aspect of the policy evaluation is to analyse the current redistribution and change of medical resources and the impact on the safety of the medication.

The purpose of a drug utilization review is to seek rational guidance for clinical medication. This rationalization not only refers to evaluating the effect of preventing and treating diseases from the medical side but also refers to evaluating its rationality regarding the society and economy to obtain the greatest social and economic benefits. Before and after each step of healthcare reform in China, there have been changes in the quantity and types of medicines used in public hospitals, which pose new challenges to the rational use of medicines in clinical practice.

This study is based on the analysis of real-world drug utilization data, and the purpose is to define the links and influence of the specific roles of the drug policy and whether the impact of these affecting factors and modes of action on clinical drug use is in line with policy expectations. For the Essential Medicine Policy, many publications are focused on methods of evaluating institutional performance with primary hospitals as the research object [7–9]. However, in the new situation of urban public hospital reform, there is limited research on the operating mechanism and how to implement the Essential Medicine Policy at senior medical institutions. This topic starts with research on the status quo of implementation of the Essential Medicine Policy in county-level public hospitals to determine if the trend of changes in drug utilization and whether essential drugs can be prioritized in comprehensive public hospitals. In select samples and as a pilot city of public hospital reform in Anhui Province, the implementation of medical reform evaluation and development trend of research have a certain representation.

The results of this study show that there are significant differences in medicine utilization among the three sample counties, which may be related to the distinct demographic and economic situations. The top three chemical medicines with the most categories in the three samples in the past 3 years were anti-infectives (12.92%), cardiovascular drugs (11.61%) and digestive drugs (8.42%). The top three for traditional Chinese medicine were internal medicine (66.03%), surgery (8.45%) and gynaecological medicine (7.70%).

The rational use of medicine emerged from clinical pharmacy work in the 1960s. Especially, the unnecessary and inappropriate use of antibacterial drugs has always been a topic and challenge of global concern from then on until now [10]. Although chronic non-infectious diseases, from a disease classification perspective, predominantly affect country areas in Anhui province, however, the use of anti-infectives is much higher than other types of drugs. In addition, it has caused our great concern that the usage of broad-spectrum antibiotics is much higher than narrow-spectrum antibiotics and other anti-infectives. Combined with the results of field interviews and experts’ consultation, anti-infective drug should be chosen to implement a long-term drug monitoring program, and clinical usage of anti-infective medicines require standardization along with further pharmacovigilance monitoring. For other results and according to the statistics of dosage forms, the proportion of Chinese medicine injections increased from 2011 to 2013, and the increase was statistically significant (***χ***^***2***^ = 28.428, *P* < 0.01). The injection of Chinese traditional medicine has been widely used to treat various diseases in the past few decades. Compared with the traditional oral administration form, injection administration is basically superior in terms of both biological availability and therapeutic effects. However, its security also caused a growing concern due to the complicated constituents and the intricate mechanism of action. Chinese traditional medicine injection has already been listed as one of the clinical monitoring drugs, clinicians should carefully consider the balance between its safety and therapeutic effects.

Combining the above results, whether there is a difference between the regions or the difference concerning time, a series of questions have been raised. For example, can the implementation of the NEML improve the public accessibility and availability of drugs? Is the intention of the application of NEML to provide cheaper medicines or benefit clinical rational drug use?

Rational drug utilization refers to when medical personnel explicitly carry out individualized medication treatment with contemporary, systematic, and comprehensive medical, pharmaceutical and management knowledge in the process of prevention, diagnosis, and treatment of disease to guide medication for individual patients [11]. There is a universal puzzle worldwide—has a large amount of drug consumption correspondingly improved people’s health? Since there is a certain gap between the developing countries (who have distinct capabilities and needs) and first world countries, the solutions of developing countries for problems regarding rational drug utilization are mainly public education and stringent regulations. Interventions should be conducted in accordance with the four elements of rational utilization of medication defined by the WHO, namely, safe, effective, economical and appropriate [12]. There are many factors that affect the rational use of drugs in different phases and in different regulatory environments for pharmaceutical management. The key factors are the substantial implementation of National Medicine Policy, the standardization of medical practices, and the enhancement of pharmaceutical services.

Of the rational drug use interventions, the essential medicine policy for rational drug use in developing countries was proposed by the WHO at the 28th World Health Assembly in 1975 [13]. As a result, the selection of the essential medicines list and other lists has led to the development of a global study on how to choose medicines under different national conditions to ensure that people have access to medications for common diseases [14].

In China, NEML, BWM ML and NRCM ML are the major lists that promote the implementation of national medicine policies and guarantee the health and insurance benefits of urban workers, residents and farmers. NEML takes the national conditions of both the world and China into account and includes essential chemical and Chinese drugs [15, 16]. Thus, the production and supply of drugs in this list should be guaranteed. BWM ML emphasizes the connection between the payment and salary level, affordability and disease burden, which is the most practical booster for NEML. At the same time, BWM ML should gradually improve and perfect the NRCMML. However, all of the current lists (3 totals) have ignored the opinions from health agencies, health service providers and patients. Therefore, different medicine lists made by different departments based on distinct aims are open for use at the same time for the same patient cohorts, which harm the outcomes of each list in practice.

The results of this study show that clinical medication for three consecutive years, in the six county hospitals, and classified by medicine list demonstrates that the medicines used and belonging to BWM ML (counted by categories) account for 94.37% of the total. The proportion of NEML is 48.48%. These data suggest that clinicians at county-level health care institutions prefer to prescribe medications in the Medical Insurance Medicine List and are less concerned with the clinical rational prioritization of essential medicines. When the sales amount is calculated, the proportion of BWM ML is 88.93% and the proportion of NEML is 38.8%. There are two reasons for the sales of essential medicines accounting for only a small proportion: one is that most drugs used in medical institutions are not essential drugs, and the other is that the price of essential medicines is generally low. It is not difficult to observe that the proportion of the medical insurance list far overweighs the NEML in terms of both sales amount and quantity. It is worth noting that the proportions of the medicines, which are counted by categories, in both the NEML and BWM ML show no significant difference between 2011 and 2012 (BWM ML: ***χ***^***2***^ = 1.069, *P* = 0.301; NEML: ***χ***^***2***^ = 1.324, *P* = 0.250). However, when comparing the 2011 and 2013 and 2012 and 2013, we observed significant differences. Therefore, the changes in drug utilization in 2013 may be the major part of the changes in the overall composition. Since January 2013, all of the drugs used in 148 county hospitals in 74 pilot counties (cities and districts) in Anhui Province have been sold at zero-profit, which may be the main reason for the changes.

For the purpose of giving essential medicine policy full implementation to guarantee the drug supply and promotion of rational drug utilization, medical personnel need to raise awareness of the essential medicine policy. The reality is that the essential medicines in the county hospitals get far less emphasis than medical insurance medications. Hence, this is a long way from the objective of medical reform, which aims at propelling essential medicines as a priority to solve clinical problems [17]. Anhui Province has initiated integration of the NRCMR ML and BWM ML even though problems of the application of the united comprehensive medicine list remain to be solved. Meanwhile, how to smoothly integrate essential medicine policy with the national basic medicine insurance and reimbursement system is another important task in the next stage of Anhui reform. In this way, equitable distribution of medical resources can occur, and individuals could enjoy basic healthcare security [18].

## Conclusion

This study uncovered the changing tendency of drug utilization in county-level hospital under the implementation of the reform. The results indicate that comprehensive measures of medical reform have had a remarkable impact on the actual utilization of medicines. However, compared with the drug included in Basic National Medical Insurance List, essential medicines are not so prioritized used as expected. Strategies to improve physicians’ intention to preferentially prescript essential medicines should focus on helping individuals to recognize the value of NEMP, establishing information communicating mechanism for NEMP.

In view of the high frequency use of injections of traditional Chinese medicines and anti-infectives, explicit classification of drug administration, dynamic monitoring and continuous rational use improvement are needed. Meanwhile, varied evidence of rational drug utilization should be provided from different perspectives of clinical practice.

### Limitation

This study focused on the trend of drug utilization over a period of time. It has some limitations. First, since most of these indicators did not relate the diagnosis to the disease, the results cannot tell us exactly what proportion of the people were treated correctly or the exact nature of the drug use problem; the results can only indicate that there might be a drug use problem. Second, different disease patterns and prescriber types will greatly affect the indicators, therefore, advanced analysis should be conducted by diagnosis or prescriber type if these vary between the compared facilities. Furthermore, this study focused on only one province. This population-based analysis should be conducted in more areas and more hospitals should be included. We are planning more widespread investigations in further studies.

## Abbreviations

BWM ML: National Basic Medical Insurance Work-Related Injury Insurance and Maternity Insurance Medicine List
HIS: Hospital Information System
NEML: National Essential Medicine List
NEMP: National Essential Medicine Policy
NRCMML: New Rural Cooperative Medical Reimbursement Medicine List
SPSS: Statistical Package for the Social Sciences
WHO EML: World Health Organization Essential Medicine List

## References

[1] Communist Party of China Central Committee. State Council. Notice on the issuance of the opinions on the establishment of National Essential Medicine System. Beijing: Government of the People's Republic of China; 2009.

[2] Zhang Q, Liu LP, Wang YY, Xie XF (2015). Analysis of the use of antimicrobial agents in a hospital from 2012 to 2013. Anhui Med Pharma J.

[3] Wu AH, WEn XM, Li CH, Ren N, Gong RE, Huang X, Feng L, Liu ZR, MEng L, Guo YH (2014). The national hospital infection rate and cross-section antimicrobial drug usage report in 2012. Chin J Infecti Control.

[4] Lin H, Dyar OJ, Rosales-Klintz S, Zhang J, Tomson G, Hao M, Stalsby Lundborg C (2016). Trends and patterns of antibiotic consumption in Shanghai municipality, China: a 6-year surveillance with sales records, 2009-14. J Antimicrob Chemother.

[5] The Anhui Department of Health. Notice concerning the priority of the use of essential medicines in medical institutions above county level. Hefei: The Anhui Provincial Government; 2011.

[6] MNGD, Fu Y. Drug use research - methods and applications. Wuhan: Chinese Journal of Pharmacoepidemiologyed; 1998.

[7] Yang L, Liu C, Ferrier JA, Zhou W, Zhang X (2013). The impact of the National Essential Medicines Policy on prescribing behaviours in primary care facilities in Hubei province of China. Health Policy Plan.

[8] Yip WC, Hsiao WC, Chen W, Hu S, Ma J, Maynard A (2012). Early appraisal of China’s huge and complex health-care reforms. Lancet.

[9] Li L, Ye L (2012). The effect of medicine zero profit policy on the drug expenses in primary health centers. Chin J Health Econ Res..

[10] Zhang ZH (2016). The present situation and development trend of clinical pharmacy service in county hospital. J Clinical Med Liter.

[11] Zhou W (2007). Pharmacoepidemiology.

[12] WHO. The World Medicines Situation: Rational use of Medicines. 2004. http://apps.who.int/medicinedocs/en/d/Js6160e/10.html.

[13] WHO. Indicators for monitoring national drug policies. A practical manual. Second edition. Geneva: World Health Organization; 2000.

[14] Seuba X (2006). A human rights approach to the WHO Model List of Essential Medicines. Bull World Health Organ.

[15] Millard C, Brhlikova P, Pollock A (2015). Social networks and health policy: the case of misoprostol and the WHO model essential medicine list. Soc Sci Med.

[16] Ye L (2008). National Essential Medicine Policy Research.[D].

[17] Cui L, Li XS, Tian J, Zhang J, Yang LJ, Yang HB, Yu L, Xie R (2011). Investigation on the cognition of the National Essential Medicine System by the primary medical staff. Chin Pharm.

[18] The Anhui Provincial Government. Notice on the comprehensive reform of medical and health system in Anhui province. http://www.ah.gov.cn/UserData/DocHtml/1/2015/4/22/4159085957250.html.

